# The Orexin-A-Regulated Akt/mTOR Pathway Promotes Cell Proliferation Through Inhibiting Apoptosis in Pancreatic Cancer Cells

**DOI:** 10.3389/fendo.2018.00647

**Published:** 2018-10-31

**Authors:** Linna Suo, Xiaocen Chang, Yuyan Zhao

**Affiliations:** ^1^Department of Endocrinology, The First Affiliated Hospital, China Medical University, Shenyang, China; ^2^Department of Endocrinology and Metabolism, The Fourth Affiliated Hospital, China Medical University, Shenyang, China

**Keywords:** orexin-A, OX1R, Akt, mTOR, apoptosis, pancreatic cancer

## Abstract

The orexin-A and its receptors are associated with many physiological processes in peripheral organs and the central nervous system and play important roles in a series of human diseases, including narcolepsy, obesity, and drug addiction. Increasing evidence has indicated high expression of orexin-A and OX1 receptor (OX1R) in malignant tumors, suggesting that the stimulation of OX1R might be essential for tumorigenesis. Here, we attempted to clarify the correlation between orexin-A expression and malignancy in pancreatic cancer. Our results indicated that the stimulation of OX1R promotes cell proliferation in pancreatic cancer PANC1 cells. Additionally, orexin-A treatment can protect PANC1 cells from apoptosis, whereas inhibition of the stimulation of OX1R results in apoptosis through regulating pancreatic cancer cell expression levels of Bcl-2, caspase-9, and c-myc, which are key apoptotic factors. Further investigation revealed that orexin-A treatment activates theAkt/mTOR signaling pathway to promote cell proliferation byinhibiting Bcl-2/caspase-9/c-myc-mediated apoptosis in pancreatic cancer cells. Our findings revealed that the stimulation of OX1R might be important for tumorigenesis in pancreatic cancer and is a potential target for the treatment of patients with pancreatic cancer.

## Introduction

Pancreatic cancer (PC), a relatively rare but highly lethal malignancy, is projected to be the third most deadly cancer, and 53,670 estimated cases and 43,090 deaths occurred in the United States in 2017 (1). The advances in the treatment of this disease have been slow, and the current 5 year survival rate is only 8% (1). The poor prognosis related to pancreatic cancer is due to delayed diagnosis because over 90% of patients with pancreatic cancer are diagnosed at stages III or IV (2). Therefore, the identification of novel biomarkers responsible for the progression of pancreatic cancer might be required for the prevention, early diagnosis and improved systemic therapy of pancreatic cancer.

The neuropeptides orexin-A And orexin-B, both derived from the proteolytic cleavage of the precursor molecule prepro-orexin (3, 4), are exclusively secreted by neurons in the lateral and posterior hypothalamic areas (5), which project to other brain regions, including the stem nuclei and thalamus, forebrain region and spinal cord (6–8), and play important roles in the regulation of feeding behavior, sleep/wakefulness cycles, reward system, energy homeostasis and other physiological processes by activating various downstream pathways (9–13). The functions of orexin peptides are mediated via interaction with two G-protein-coupled receptors (GPCRs), orexin receptor 1 (OX1R) and orexin receptor 2 (OX2R) (4, 14, 15). OX1R has a higher selectivity for orexin-A, whereas OX2R usually binds both orexin peptides with similar affinity (14). In addition to the hypothalamus, orexins and their receptors are widely distributed throughout peripheral tissues, including adipose, gut, pancreatic, adrenal gland and testis tissues (16–18).

Recent studies have indicated that theorexin-A/OX1R signaling cascade is associated with progression in colon cancer, prostate cancer, gastric cancer, and hepatocellular carcinoma. Previous reports have shown that the stimulation of OX1R is involved in progression in prostate cancer (19). Further investigation demonstrated that thestimulation of OX1R can antagonize testosterone-induced androgen receptor nuclear translocation, suggesting that orexin-A might play a role in the regulation of androgen/androgen receptor signaling in prostate cancer (20). Bai et al. showed that OX1R can regulate migration in human colon cancer cells (21). Additionally, a recent study indicated that orexin-A can induce autophagy in HCT-116 human colon cancer cells through the ERK signaling pathway (22). Furthermore, several studies also indicated that stimulation of OX1R can mediate glucose metabolism through stimulating HIF-1α signaling and PI3K/Akt/mTOR pathways in human hepatocellular carcinoma cells (23, 24), suggesting that orexin-A/OX1R might be a potential target for malignant cancer therapy. However, the possible roles of the orexin-A/OX1R signaling cascade are largely unknown in pancreatic cancer.

In the present study, we investigated the correlation between the stimulation of OX1R and progression in pancreatic cancer. Our results showed that the stimulation of OX1R may be associated with malignancy in pancreatic cancer. Additionally, we found that orexin-A treatment can promote cell proliferation through inhibiting Akt/mTOR-mediated apoptosis in pancreatic cancer PANC1 cells. Our findings provide insight into the mechanisms by which orexin-A/OX1R contributes to cell proliferation and apoptosis, which might be helpful for clinical therapy for patients with pancreatic cancer.

## Materials and methods

### Antibodies and reagents

Mouse monoclonal anti-orexin-A antibody (sc-80263) and goat polyclonal anti-orexin receptor-1 antibody (sc-8072; 1:2,000) were obtained from Santa Cruz Biotechnology (Santa Cruz, CA, United States); rabbit polyclonal anti-prepro-orexin antibody (ab3096; 1:2,000), rabbit monoclonal anti-phospho-mTOR antibody(ab109268; 1:2,000), and rabbit monoclonal anti-caspase-9 antibody(ab32539; 1:2,000) were from Abcam company (Cambridge, United Kingdom); mouse monoclonal p-Akt antibody (66444-1; 1:2,000), mouse monoclonal Akt antibody (60203-2; 1:2,000), rabbit polyclonal anti-mTOR antibody (20657-1; 1:2,000), rabbit polyclonal anti-Bcl-2 antibody (12789-1; 1:2000), and rabbit polyclonal anti-c-Myc antibody (10828-1; 1:2,000) were purchased from ProteinTech Group Inc. (Rosemone, IL, United States); anti-GAPDH antibody (100242; 1:5,000) was purchased from Sino Biological Inc. (Beijing, China). The secondary antibodies were from Jackson ImmunoResearch Laboratories Inc. (1:5000; West Grove, PA, United States). Orexin A was obtained Sigma-Aldrich (St. Louis, MO, United States). The OX1R antagonist, SB408124 was from Tocris Bioscience (Bristol, United Kingdom); the inhibitor of the Akt pathway, MK-2206, was obtained from Selleck Company (Houston, TX, United States).

### Cell culture

The pancreatic cancer PANC1 cell line and normal pancreatic HPC-Y5 cell line were obtained from American Type Culture Collection (Manassas, VA, United States) and were maintained in DMEM medium (DMEM; Gibco, Thermo Fisher Scientific, Waltham, MA, United States) supplemented with 10% fetal bovine serum (FBS; Gibco), 50 μg/ml of penicillin and 100 μg/ml of streptomycin (Invitrogen, Thermo Fisher Scientific) in a 5% CO_2_ humidified atmosphere at 37°C.

### Immunohistochemistry

A paraffin-embedded tissue microarray was purchased from Alenabio company (PA2072; Xi'an, China), which contained 60 cases of pancreatic cancer specimens and nine cases of normal pancreatic tissues. The slide was dewaxed with xylene and rehydrated in descending concentrations of ethanol and then incubated with anti-orexin-A antibody (1:200). After washing four times in PBST buffer for 15 min, the slide was processed according to the Super Sensitive Polymer HRP Detection System/DAB Kit protocol (Thermo Fisher Scientific, Waltham, MA, United States) and counterstained with hematoxylin. The study protocol was approved by the Ethics Committee of the Fourth Affiliated Hospital, China Medical University.

### Western blot assay

To prepare protein extracts, the cells were washed with PBS for three times. Following centrifugation, the harvested cells were resuspended and protein extracted using by lysis buffer (Pierce, Thermo Fisher Scientific) for 30 min on ice. The lysates were centrifuged at 16,000 × g for 10 min at 4°C. The supernatants of the lysates were mixed with SDS sample buffer and boiled for 10 min. The samples (~100 μg each lane) were separated by 10% SDS-PAGEelectrophoresis and then transferred to a polyvinylidenedifluoride (PVDF) membrane (Millipore, Billerica, MA, United States). The signals were evaluated using the Super Signal West Pico Substrate Kit (Pierce, Thermo Fisher Scientific) and were measured by fluorescence intensity using ImageJ software (National Institutes of Health, Bethesda, MD, United States).

### qPCR assay

Total RNA was extracted with TRIzolreagent (Invitrogen, Thermo Fisher Scientific) according to the manufacturer's instructions. qPCR was performed using a SYBR PrimeScript RT-PCR Kit (TaKaRa Bio Inc., Otsu, Japan) and the ABI 7500 real-time PCR systemby using 500 ng total RNA for each reaction. The primer sequences were as follows:prepro-orexin forward primer 5′-cgtcttgctcgagatgtgatg-3′ and reverse primer 5′-gcacacagagggctacaatgtg-3; OX1 receptor forward primer 5′-ccccactgggcctcatg-3 and reverse primer 5′-ccccagagcttgcggaata-3′; GAPDH forward primer 5′-tgcaccaccaactgcttagc-3′ and reverse primer 5′-ggcatggactgtggtcatgag-3′. Each sample was replicated three times.

### Cell proliferation assay

The cell proliferation assay was performed using acell counting kit (CCK-8 Kit) (Beyotime, Beijing, China). Briefly, all cells were seeded in 2,000 cells/well in phenol red-free DMEM medium with 10% FBS in a 96-well plate. A total of 10 μl of CCK-8 working solution was added in each well from days 1 to 5, followed by incubation for 2 h at 37°C, and the absorbance was finally measured at 450 nm.

### Colony formation assay

To a 24-well plate, 5 × 10^2^ orexin-a-treated (10^−7^ M) cells with or without SB408124 (50 nM) treatment were added. Then, all cells were cultured for 2 weeks with fresh medium replacement every 3 days. Cells were stained with crystal violet for 10 min and weredestained with PBS three times. Colonies were counted using ImageJ software for each well, and triplicate repeats were performed for each condition.

### Apoptosis assay

Hoechst 33342 staining (Beyotime) was performed to examine cell apoptosis in the study. Briefly, all the cells were seeded on coverslips in a 24-well plate and were cultured for 24 h. After removing the cell culture medium and washing the cells three times, the cells were incubated in the Hoechst labeling solution (2 μg/ml) for 20 min at room temperature. The cells were then washed with PBS three times and were observed under a fluorescence microscope.

### Statistical analysis

The results were expressed as the means ± standard error of the mean, and differences between the means were analyzed by one-way or two-way analysis of variance (ANOVA) where appropriate. *P* < 0.05 was considered to be statistically significant. Statistical analysis was performed using SPSS statistical software (SPSS Inc., Chicago, IL, United States).

## Results

### Increased orexin-A level in advanced human pancreatic cancer tissues

**Figure 1:**
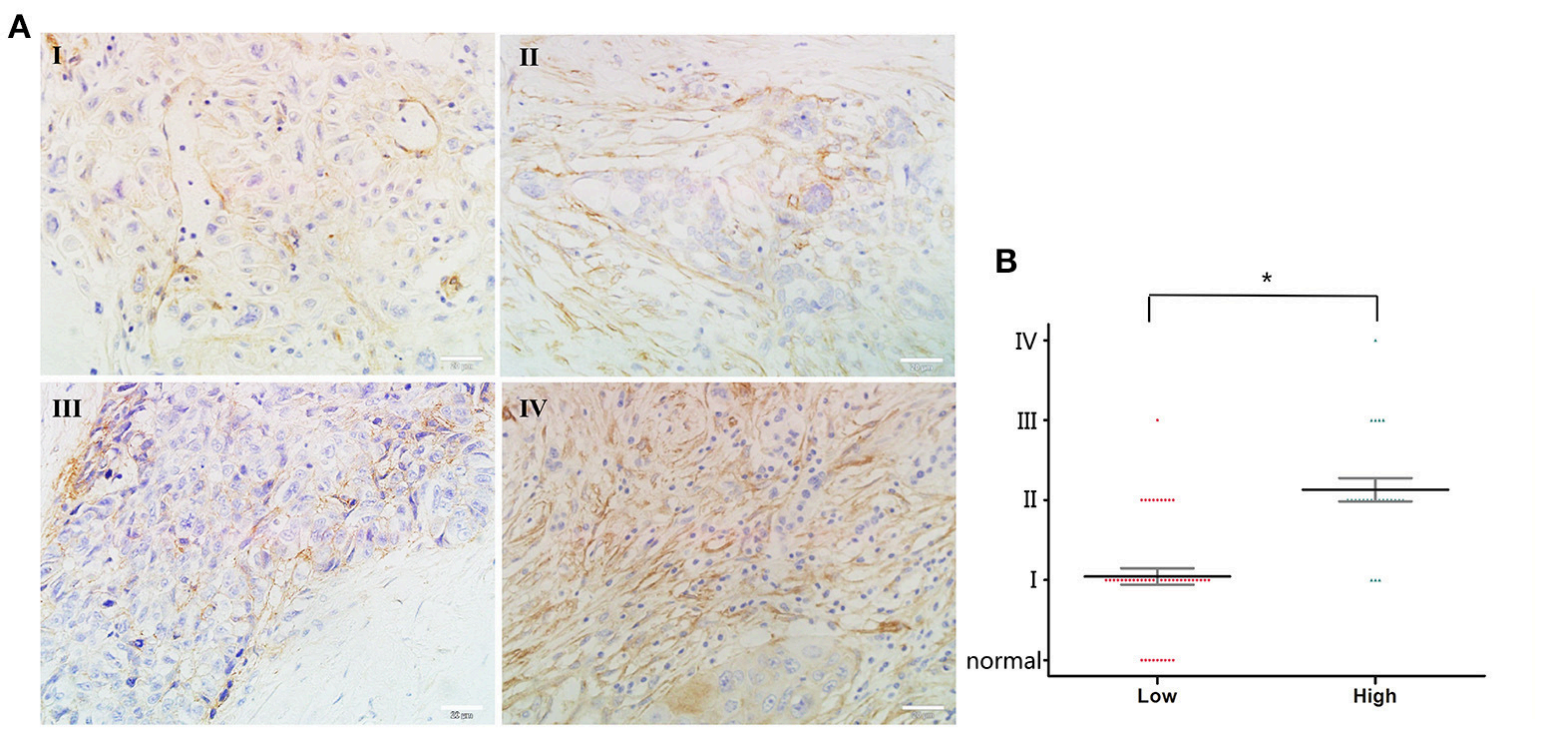
Correlation between orexin-A expression and malignancy in pancreatic cancer **(A)**. Representative images of orexin-A expression in pancreatic cancer at different stages **(B)**. Pathological analysis for correlation between orexin-A expression and malignancy in pancreatic cancer. *p < 0.05; Scale bars, 20 μm in **(A)**.

Previous reports have indicated that orexin-A expression might be associated with malignancy in several tumors (20, 23, 24). Therefore, we examined the potential functions of orexin-A in human pancreatic cancer. First, we performed immunohistochemical analysis of orexin-A expression in a commercial microarray of 60 human pancreatic cancer specimens and 9 normal/adjacent pancreatic tissues (Table 1). Based on the overall staining intensity, Figure 1A shows that orexin-A immunostaining was weak in pancreatic cancer specimens (stage I and II), whereas a high expression level of orexin-A was observed in pancreatic cancer specimens (stages III and IV), indicating that the expression level of orexin-A might be associated with malignancy in the patients with pancreatic cancer. Further quantitative analysis indicated that the upregulation of orexin-A is proportional to the stage of malignancy in pancreatic cancer tissues and might have functional relevance (Figure 1B).

**Table 1:** Characteristics of patients with pancreatic cancer.

**Characteristic**	**Adenocarcinoma (*n* = 60)**	**Normal tissues (*n* = 9)**
**AGE (YEARS)**		
≥60	23 (38%)	0
< 60	37 (62%)	9
**GENDER**		
Male	36 (60%)	5
Female	24 (40%)	4
**GRADES**		
1–2	37 (62%)	-
3–4	23 (38%)	-
**STAGES**		
I	30 (50%)	-
II	24 (40%)	-
III+IV	6 (10%)	-

### The stimulation of OX1R is involved in cell proliferation in PANC1 cells

**Figure 2:**
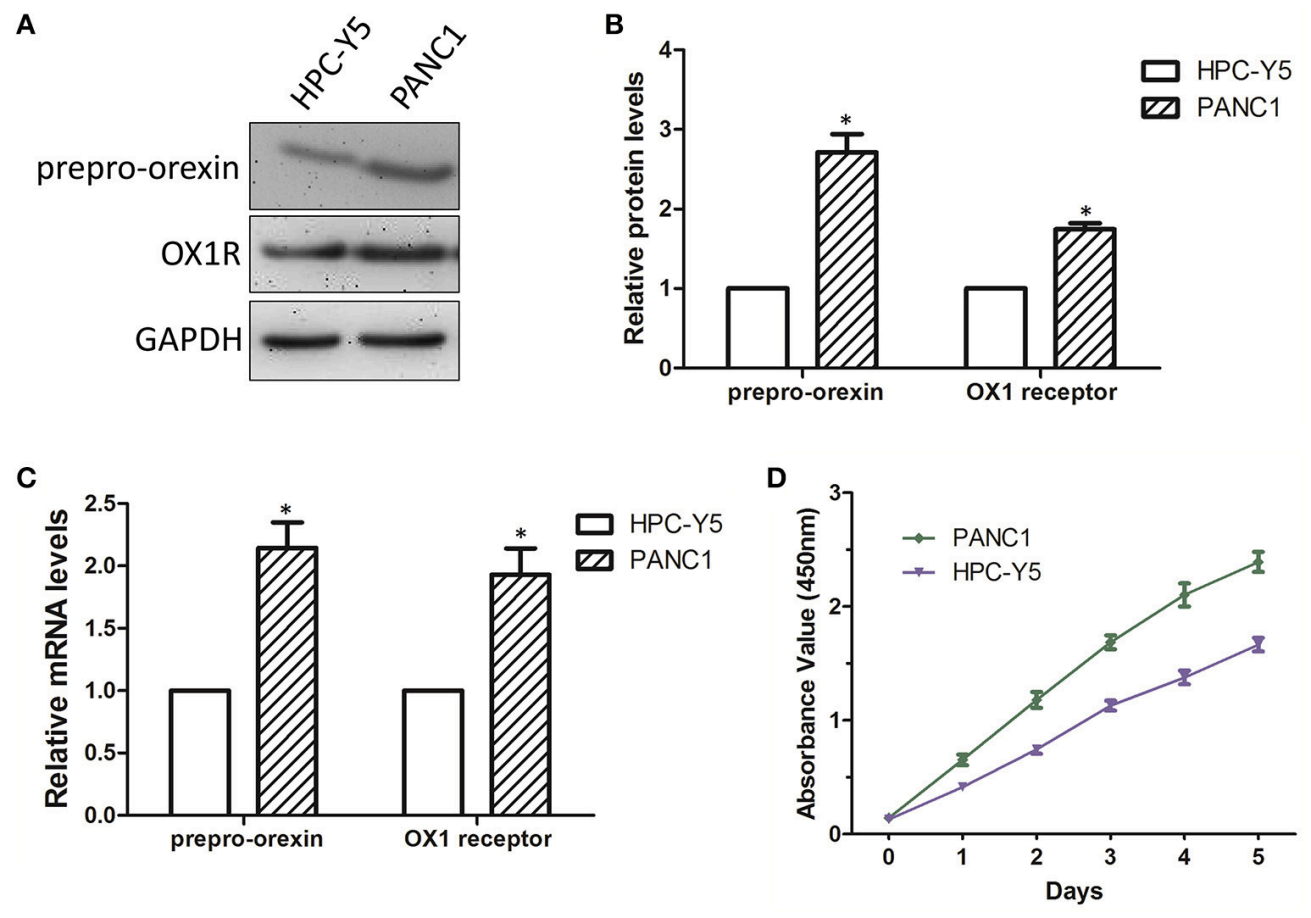
Determination of cell proliferation in PANC1 and HPC-Y5 cell lines **(A)**. Expression levels of prepro-orexin and OX1 receptor in HPC-Y5 and PANC1 cell lines;Quantitative analysis of the expression and mRNA levels of prepro-orexin and OX1 receptor using western blot **(B)** and qRT-PCR assays **(C,D)** Cell proliferation of PANC1 and HPC-Y5 cell lines. *p < 0.05, compared with the HPC-Y5.

To further investigate the role of orexin-A and its receptor in cell proliferation in pancreatic cancer cells, we next examined the expression levels of theorexin-A precursor molecule prepro-orexin and OX1R in PANC1 and HPC-Y5 cell lines by western blot analysis and qRT-PCR. We found that the expression levels of prepro-orexin and OX1R in PANC1 cells were higher than those in HPC-Y5 cells (Figures 2A,B). Similarly, theqRT-PCR assay showed over 2-fold expression levels of prepro-orexin and OX1R in PANC1 cells (Figure 2C). This evidence indicated the high expression of either prepro-orexin or OX1R in pancreatic cancer PANC1 cells, suggesting that thestimulation of OX1R might play a role in tumorigenesis in pancreatic cancer. Furthermore, we examined the cell proliferation between the pancreatic cancer PANC1 cells and normal pancreatic HPC-Y5 cells. Our results showed that the cell proliferation in PANC1 cells was much higher than that in HPC-Y5 cells (Figure 2D). Therefore, we expected that the stimulation of OX1R may be associated with cell proliferation in pancreatic cancer PANC1 cells.

### Orexin-A treatment induces cell proliferation in PANC1 cells

**Figure 3:**
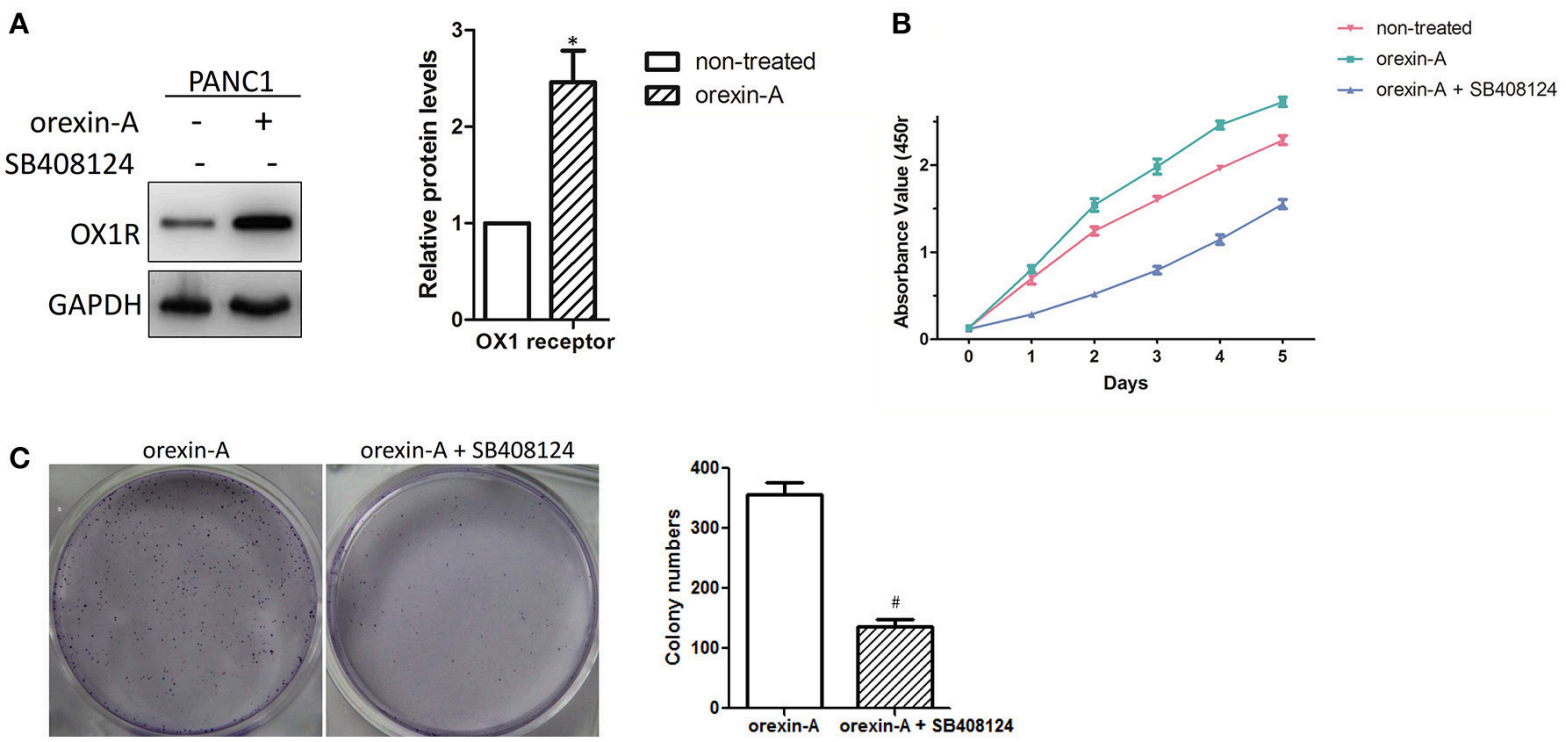
Orexin-A promotes cell proliferation in pancreatic cancer cells **(A)**. Determination of OX1 receptor expression in 10^−7^ M orexin-A-incubated PANC1 cells **(B)**. Determination of the cell proliferation of 10^−7^ M orexin-A-incubated PANC1 cells with or without SB408124 treatment **(C)**. Representative images and statistical analysis of colony formation in 10^−7^ M orexin-A-incubated PANC1 cells with or without SB408124 treatment. *p < 0.05, compared with control (non-treated); ^#^*p* < 0.05, compared with orexin-A.

To determine the biological functions of orexin-A in pancreatic cancer, we activated or inactivated the stimulation of OX1Rby incubation with different concentrations (10^−5^, 10^−6^, 10^−7^, and 10^−8^ M) of orexin-A with or without treatment of SB408124 (50 nM), an OX1 receptor antagonist to prevent the orexin-A effect on cell proliferation in PANC1 cells (data not shown). We found that treatment with10^−7^ M orexin-A can significantly upregulate OX1R expression in PANC1 cells (Figure 3A), which is consistent with that of previous reports (20, 23).

Due to the different growth rates between PANC1 and HPC-Y5 cells, we further studied the functions of orexin-A in cell proliferation in pancreatic cancer cells. Then, the proliferation of the orexin-A-incubated PANC1 cells with or without treatment with 50 nM SB408124 was measured. The proliferation of PANC1 cells treated with 10^−7^ M orexin-A was much higher than that of non-treated PANC1 cells, whereas cell proliferation, which was induced by orexin-A incubation, was remarkably inhibited by SB408124 treatment in PANC1 cells (Figure 3B). Moreover, compared with orexin-A-treated PANC1 cells, treatment withorexin-A and SB408124 resulted in the inhibition of colony formation in PANC1 cells (Figure 3C), suggesting that orexin-A treatment can significantly promote cell proliferation in pancreatic cancer cells.

### Orexin-A-mediated cell proliferation occurs through inhibiting cell apoptosis in pancreatic cancer cells

**Figure 4:**
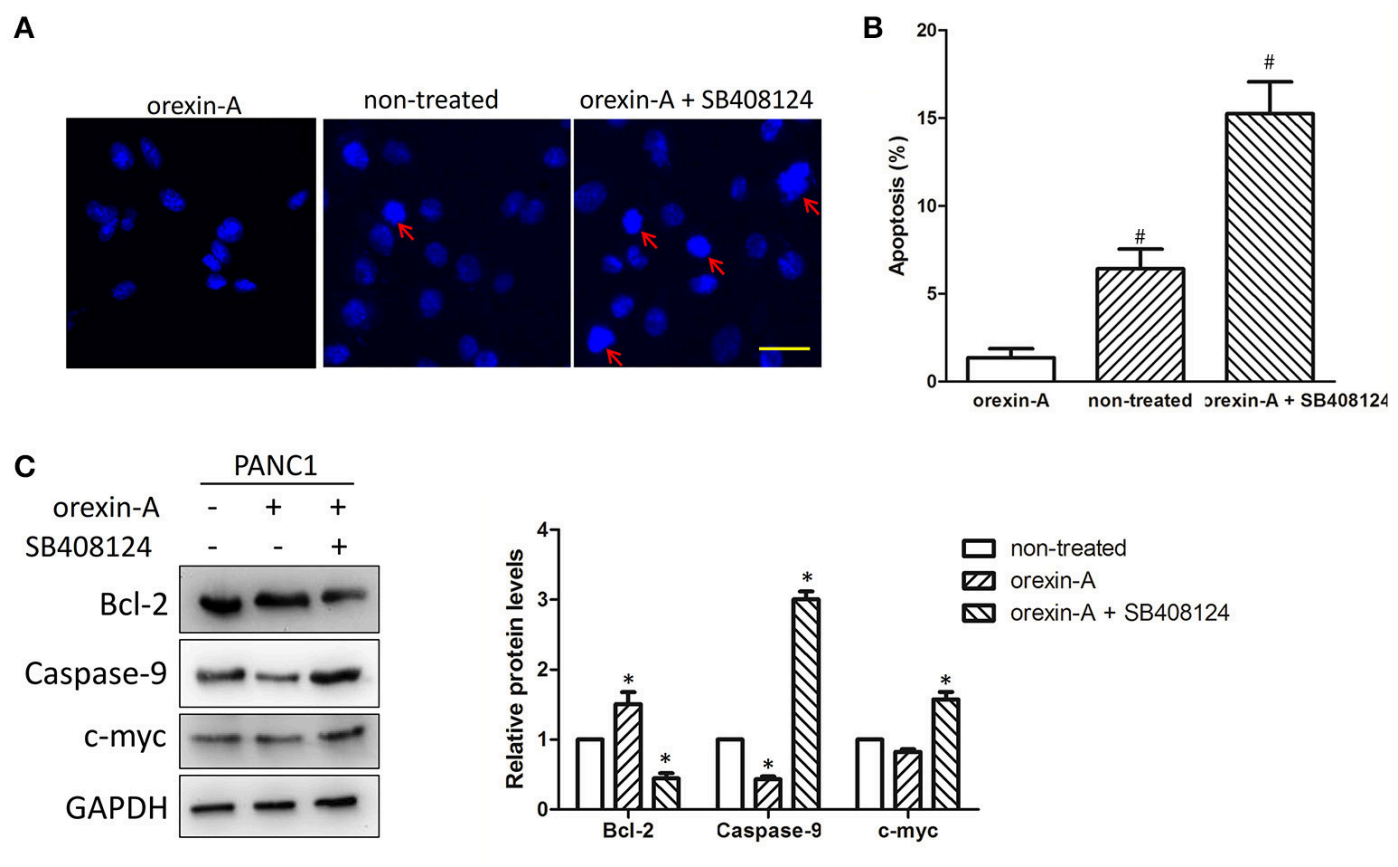
Orexin-A treatment promotes cell proliferation through inhibiting apoptosis in pancreatic cancer cells **(A)**. Representative images of Hoechst-stained cells in 10^−7^-M orexin-A-incubated PANC1 cells with or without SB408124 treatment **(B)**. Percentage of apoptotic cells in 10^−7^-M orexin-A-incubated PANC1 cells with or without SB408124 treatment **(C)**. Expression levels of the OX1 receptor, Bcl-2, caspase-9, c-myc in 10^−7^-M orexin-A-incubated PANC1 cells with or without SB408124 treatment. *p < 0.05, comparedwith the control (non-treated); ^#^*p* < 0.05, compared with orexin-A; Scale bars, 20 μm.

Because cell apoptosis plays an important role in the regulation of cell proliferation in various malignant tumors, we expected that orexin-A treatment may promote cell proliferation through inhibiting cell apoptosis in pancreatic cancer cells. To illustrate orexin-A functions in cell proliferation in pancreatic cancer cells, we performed Hoechst staining to confirm whether regulation of the stimulation of OX1R was involved in the induction of cell apoptosis in orexin-A-incubated PANC1 cells with or without SB408124 treatment. Our results indicated that SB408124 treatment, which subsequently resulted in the inhibition of stimulation of OX1R, induced cell apoptosis in PANC1 cells (Figures 4A,B, right panel, red arrow), whereas cell apoptosis was almost undetectable in orexin-A-treated or non-treated PANC1 cells (Figures 4A,B, left and middle panels, red arrow), suggesting that the regulation of the stimulation of OX1R may be involved in cell apoptosis in pancreatic cancer cells.

To further investigate the possible mechanism of orexin-A-inhibited apoptosis in pancreatic cancer cells, we next examined the expression of Bcl-2, caspase-9, and c-myc proteins, which are key factors in cell apoptosis (25–27), in orexin-A-incubated PANC1 cells with or without SB408124 treatment. Notably, we found that orexin-A treatment not only upregulated the expression level of OX1R (Figure 3A), but also significantly upregulated Bcl-2 expression and downregulated caspase-9 expression. Additionally, the regulation of c-myc expression was not obvious in orexin-A-treated PANC1 cells. By contrast, SB408124 treatment, which significantly inhibits the stimulation of OX1R, can downregulate Bcl-2 expression, and upregulatethe expression level of caspase-9 or c-myc protein (Figure 4C). Therefore, our results indicated that orexin-A-mediated cell apoptosis occurs through regulating Bcl-2, caspase-9, and c-myc expression in pancreatic cancer cells.

### The Akt/mTOR pathway is associated with orexin-a-induced cell proliferation through regulating apoptosis in pancreatic cancer cells

**Figure 5:**
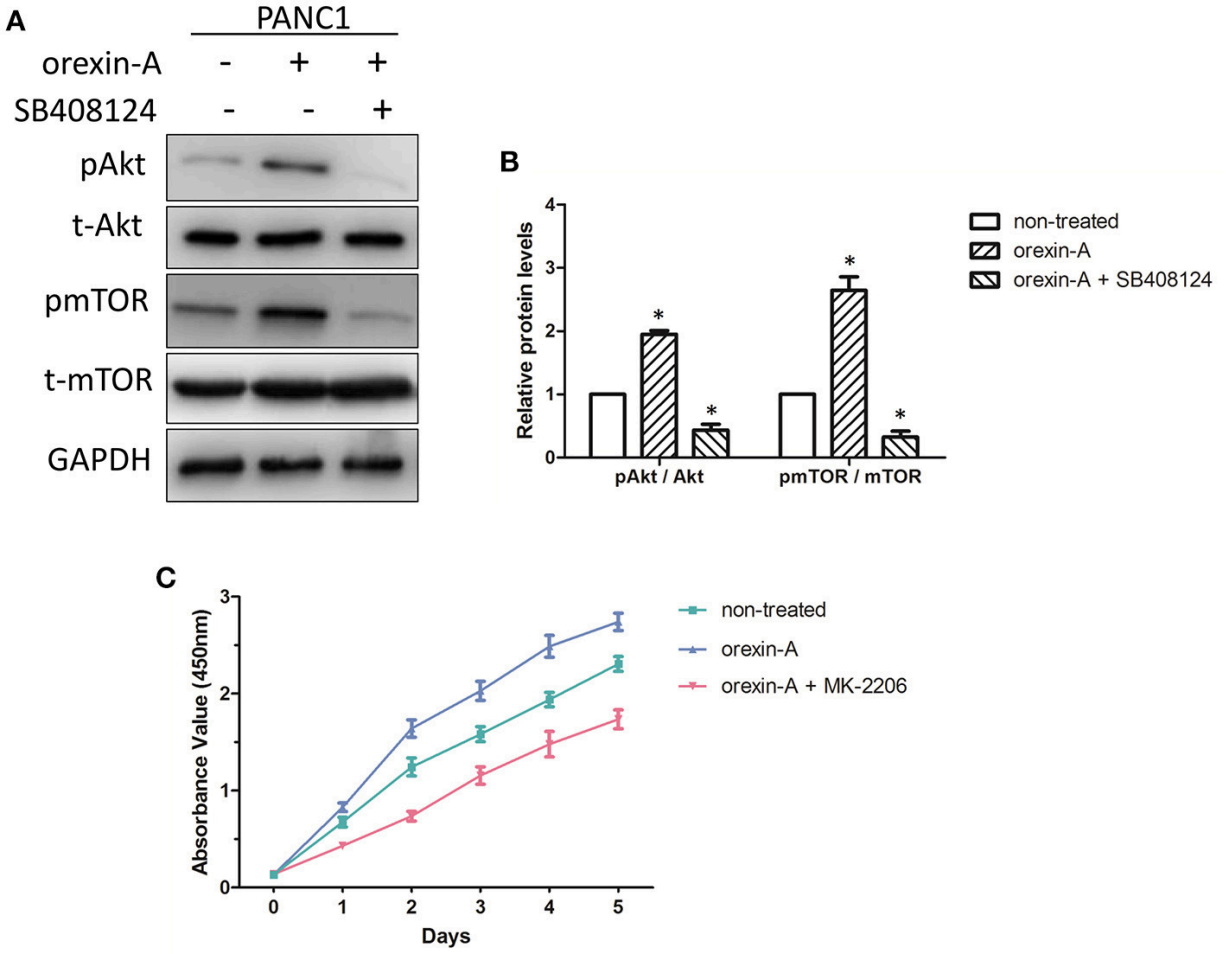
The Akt/mTOR pathway is associated with orexin-A-mediated cell proliferation through regulating apoptosis in pancreatic cancer cells **(A)**. Expression levels of the OX1 receptor, pAkt, t-Akt, pmTOR, and t-mTOR in 10^−7^-M orexin-A-incubated PANC1 cells with or without SB408124 treatment **(B)**. Activation of the Akt/mTOR signaling pathway in orexin-A-incubated PANC1 cells **(C)**. Cell proliferation of 10^−7^-M orexin-A-incubated PANC1 cells with or without MK-2206 treatment. pAkt, phosphorylated Akt; t-Akt, total Akt; pmTOR, phosphorylated mTOR; t-mTOR, total mTOR. *p < 0.05, compared with the control (non-treated).

Previous studies have described that orexin-A treatment can regulate theAkt/mTOR pathway in malignant tumors or tissues (24, 28), encouraging us to clarify the mechanism of orexin-A-regulated cell proliferation in pancreatic cancer cells. Bcl-2 and caspase-9 are the key factors of cell apoptosis, which is also regulated by the Akt/mTOR signaling pathway (29). Additionally, the Akt/mTOR signaling pathway was also involved in c-myc-induced apoptosis in various tumors (30, 31). Thus, we examined the activation of theAkt/mTOR signaling pathway in orexin-A-incubated PANC1 cells with or without SB408124 treatment. As expected, the expression levels of phosphorylated Akt and mTOR were significantly upregulated and inhibited by SB408124 treatment in orexin-A-treated PANC1 cells (Figure 5A). Additionally, quantitative analysis indicated that orexin-A treatment can activate theAkt/mTOR pathway, whereas SB408124 treatment can inhibit the activation of theAkt/mTOR pathway in PANC1 cells (Figure 5B), suggesting that theAkt/mTOR pathway may be involved in orexin-A-mediated cell proliferation through regulating apoptosis in pancreatic cancer cells.

To further confirm our hypothesis, we next examined the cell proliferation in orexin-Aincubated with or without treatment with MK-2206, an inhibitor of theAkt/mTOR signaling pathway in PANC1 cells. Interestingly, MK-2206 treatment significantly inhibited orexin-a-induced cell proliferation in PANC1 cells (Figure 5C). Therefore, this evidence suggested that orexin-A treatment may activate theAkt/mTOR pathway to inhibit apoptosis through regulating Bcl-2/caspase-9/c-myc expression and promote cell proliferation in pancreatic cancer cells.

## Discussion

Pancreatic cancer is the third leading cause of cancer-related death in western countries and is projected to be the second leading cause within a decade. Pancreatic cancer is difficult to diagnose at an early stage, with most of the cancers found to be metastatic at the time of initial diagnosis. Surgical resection is the only curative treatment for patients with pancreatic cancer, but only 15–20% of patients are candidates for pancreatectomy, which results in a poor prognosis, even after complete resection. The 5 year survival after pancreaticoduodenectomy is approximately 21% for negative margin resections and 11% for microscopically positive margin resections (32). Therefore, it is necessary to have a thorough understanding of the tumorigenesis in pancreatic cancer and identify effective biomarkers to improve the diagnostic technology for patients with pancreatic cancer. In this study, we found high expression of orexin-A in advanced pancreatic cancer specimens (Figure 1) and highexpression of prepro-orexin and OX1R in pancreatic cancer cells, resulting in cell proliferation (Figure 2) and suggesting that thestimulation of OX1R might be essential for tumorigenesis in pancreatic cancer.

It is well-known that orexins and their receptors are widely distributed throughout the brain and peripheral tissues (16, 17) and are implicated in food intake, energy homeostasis, the reward system, wakefulness, drug addiction, and neuroendocrine regulation (13, 33, 34). Recent studies have indicated that orexin-A was found to be highly expressed in prostate cancer (20) and was involved in glucose metabolism in hepatocellular carcinoma (23, 24). The present study also indicated the high expression of orexin-A in pancreatic cancer (Figure 1). We also found that orexin-A treatment can promote cell proliferation and can be inhibited by the OX1R inhibitor in PANC1 cells (Figure 3). This evidence indicated that orexin-A and its receptor, OX1R, may play an important role in tumorigenesis in pancreatic cancer.

Cell proliferation is usually associated with cell apoptosis in most malignant tumors (35, 36). Moreover, previous studies have indicated that orexin-A can protect cells from apoptosis by regulating FoxO1 and mTORC1 in hepatocytes (28), a finding that is consistent with our finding that inhibition of thestimulation of OX1R can induce apoptosis in pancreatic cancer cells (Figure 4A), indicating that the stimulation of OX1R can promote cell proliferation through inhibiting apoptosis in pancreatic cancer cells. Moreover, we also found that inhibition of thestimulation of OX1R can regulate the expression levels of Bcl-2, caspase-9, and c-myc in pancreatic cancer cells (Figure 4B). Bcl-2 is a key anti-apoptotic factor that is involved in most apoptotic processes (25). In addition, caspase-9 is usually an initiator in the apoptotic process. Zhang *et al*. found that chrysin can regulate p53/Bcl-2/caspase-9 to induce apoptosis in hepatocellular carcinoma cells (37). A recent report also revealed a unique apoptotic pathway in which antagonism of Bcl-2 family members in caspase-9-inhibited prostate cancer cells triggers caspase-8-dependent apoptosis (38). Additionally, the co-regulation of Bcl-2 and c-myc was also found to be related to cell proliferation and apoptosis in malignant tumors (27, 39). Furthermore, these three apoptosis-related proteins were key factors of the ubiquitin-proteasome system, which is an important regulator of cell growth and apoptosis (40). Therefore, we expected that the stimulation of OX1R can promote cell proliferation through regulating Bcl-2/caspase-9/c-myc-mediated apoptosis in pancreatic cancer.

Several previous studies indicated that the Akt/mTOR signaling pathway plays important roles in the regulation of cell proliferation, autophagy, and apoptosis in several malignant tumors. Want et al. found that miR-214 can regulate cell proliferation, apoptosis, and invasion via modulating the Akt/mTOR signaling pathway in cervical cancer cells (41). A previous report also demonstrated apigenin-induced autophagic apoptosis in hepatocellular carcinoma cells (42). Notably, we noticed that activation of the Akt pathway can induce apoptosis by affecting Bcl-2 and caspase-9 to regulate mouse spermatogonial cell proliferation (29). Additionally, Ladu et al. found that E2F1 may function as a critical antiapoptotic factor both in human and rodent liver cancer through its ability to counteract c-myc-driven apoptosis via activation of PIK3CA/Akt/mTOR pathways (31). We also found that inhibition of the Akt/mTOR pathway can significantly suppress cell proliferation in orexin-A-treated PANC1 cells (Figure 5C). Therefore, orexin-A can regulate apoptosis to promote cell proliferation through activating theAkt/mTOR signaling pathway in pancreatic cancer.

In summary, the data of our present study provided evidence to indicate the stimulation of OX1R can promote cell proliferation in pancreatic cancer cells. Additionally, the regulatory effect of theAkt/mTOR pathway might be associated with Bcl-2/caspase-9/c-myc-mediated apoptosis, resulting in cell proliferation in pancreatic cancer cells. Our findings revealed that orexin-A might be a potential target for the diagnosis and treatment of patients with pancreatic cancer.

## Author contributions

LS participated in the study design, performed the experimental studies and statistical analysis, interpreted the data, and drafted the manuscript. YZ participated in the study design and edited the manuscript. XC performed the experimental studies and contributed to the statistical analysis. All authors read and approved the final manuscript.

### Conflict of interest statement

The authors declare that the research was conducted in the absence of any commercial or financial relationships that could be construed as a potential conflict of interest.
